# China’s mental health support in response to COVID-19: progression, challenges and reflection

**DOI:** 10.1186/s12992-020-00634-8

**Published:** 2020-10-22

**Authors:** Yumeng Ju, Yan Zhang, Xiaoping Wang, Weihui Li, Roger M. K. Ng, Lingjiang Li

**Affiliations:** 1 Department of Psychiatry, The Second Xiangya Hospital of Central South University, Changsha, Hunan China; 2 National psychological rescue team to support for COVID-19 response in Wuhan, Wuhan, China; 3 Department of Psychiatry, Kowloon Hospital, Hong Kong, SAR China

**Keywords:** China, COVID-19, Mental health, Psychological intervention, Preparedness

## Abstract

The continued spread of the coronavirus disease 2019 (COVID-19) has a serious impact on everyone across the globe, both physically and psychologically. In addition to proactive measures addressing physical survival needs and health protection, China has launched a mental health support system to cope with the widespread psychological stress during the pandemic and its aftermath. In this debate, the authors attempted to depict and reflect upon the overall framework of China’s mental health support, with particular reference to the psychological intervention in response to COVID-19 over the last few months. Although a lot of effort has been made to meet the mental health needs, the accessibility, acceptability and effectiveness of the support system still have much room for improvement. Therefore, it is very important to re-think the predicament and challenge on ways of enhancing public mental health emergency responses in China. The concepts of universality, timeliness and scientific rigour were proposed as a possible reform in preparation for large-scale natural or man-made disasters in the coming future.

## Background

Over the last few months, the continued spreading of coronavirus disease 2019 (COVID-19) has become a “once-in-a-century” pandemic [1]. Up to August 2020, the virus has resulted in over 80 thousand confirmed cases in China and more than 20 million cases in more than 200 countries worldwide [2]. The highly contagious nature of the infection [3–5], the presence of asymptomatic carriers [6–8], and the staggering number of cases [2] trigger an unprecedented level of panic in the communities [9]. Besides the pandemic itself, stringent infection control measures issued by the government (i.e., putting cities in a lock-down state, travel bans, high restriction of people’s social activities like home quarantine and isolation), and a large number of fake news and media reports have increased emotional distress among the public [10].

**Fig. 1:**
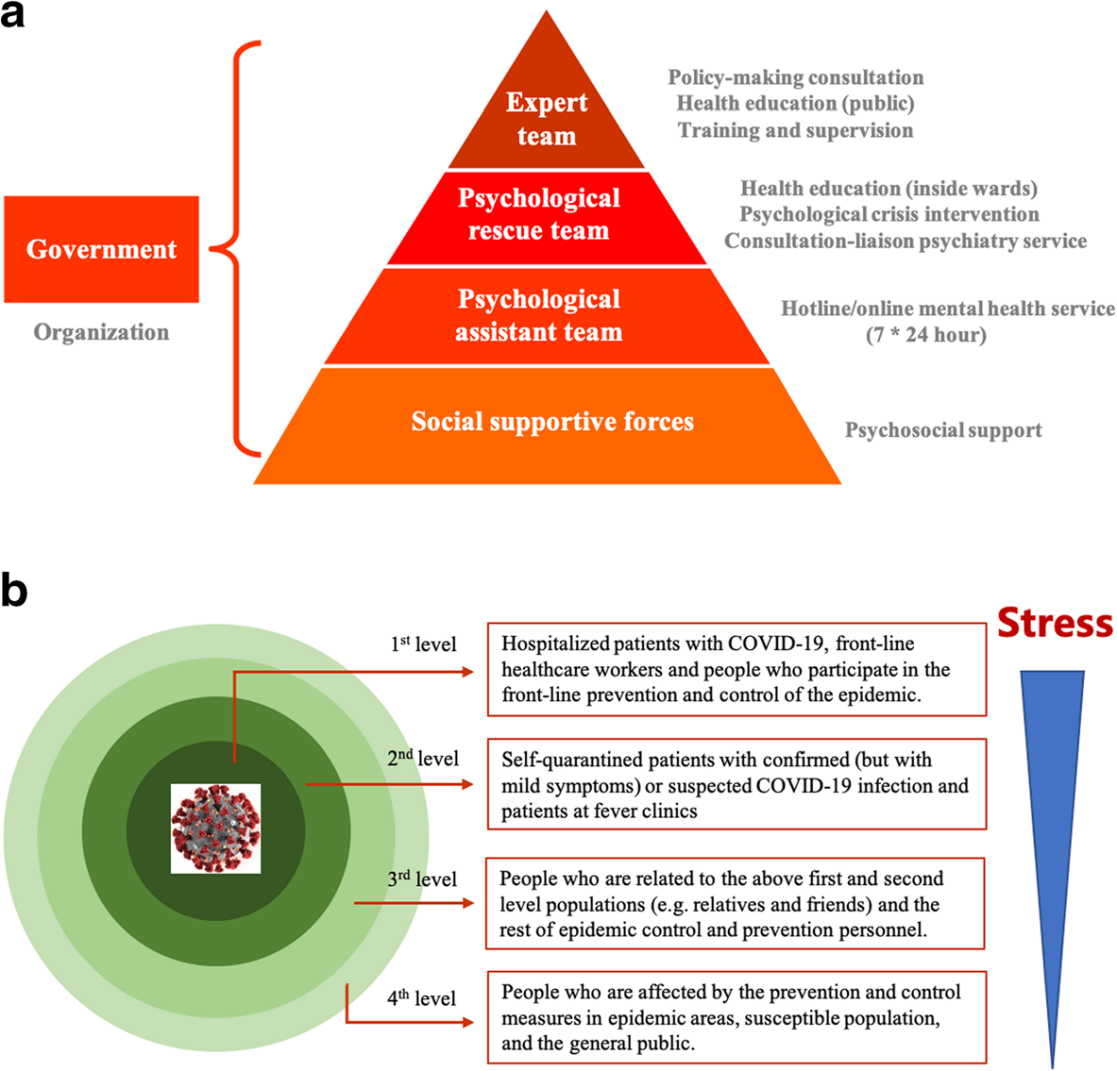
**a**. Mental health support system in response to COVID-19. In this model, the national/local government is in charge of coordination between departments. The mental health expert team consists of mental health experts in crisis intervention and has three major responsibilities: to provide policy-making consultation to the national or provincial Joint Prevention and Control Mechanism, to offer professional training and supervision for social volunteers, and to provide psychoeducation for the general public. The psychological rescue team comprises psychiatry and psychology staff as well as liaison officers. The psychological rescue team is mainly responsible for providing health education, psychological crisis intervention, consultation-liaison psychiatry service for both healthcare workers and patients inside wards. The psychological assistance team includes psychologists and professionals who are experienced in psychological crisis intervention. They take up the role of online counseling. Social supportive forces are composed of social workers, non-governmental organizations and social volunteers, who provide psychosocial support during the epidemic. **b**. Four different populations with varying levels of mental health needs according to the intensity of psychologiBBcal stressors related to COVID-19 epidemic

In face of such an unprecedented infection, on Jan 27th, 2020, China’s Joint Prevention and Control Mechanism of the State Council (China’s central authority in response to COVID-19) published the national guidance on mental health intervention with an aim to cope with the widespread mental health needs arising from this pandemic [11]. The guideline delineated a brief mental health support system and outlined the roles and responsibilities of local governments, mental health professionals, social workers, non-governmental organizations and community volunteers [12] (Fig. 1a). To enhance the effectiveness of mental health intervention for this public health emergency, the guideline also stratified the general population into four different groups with varying levels of needs according to the intensity of psychological stress experienced in relation to COVID-19 infection (Fig. 1b). Key elements of mental health interventions for the four different strata were provided in the training packages for mental health professionals. After the release of this national guidance on mental health support, China’s central health authority then issued a series of more detailed and specific official documents on mental health support [13–17]. These documents put forward concrete work plans targeting different stakeholders in the mental health support system as well as the social media. While the above policies and strategies were a significant step led by the Central Government in addressing mental health needs related to the COVID-19 pandemic, there are many challenges and opportunities during the process of implementation, both in the affected communities and in the COVID-19 designated hospitals. In the current debate, we aimed to share our observations and local work experiences on the delivery of mental health care during the pandemic. Critical reflections were also provided in this debate, with an aim to shed light on a better response to public mental health emergencies for large-scale natural or man-made disasters in the coming future.

## Mental health support in the communities

### Overview of the strategies

As people are called for quarantine during the COVID-19 epidemic, social media becomes the major mode for both information dissemination and provision of public mental health services. To date, the governmental working group and the mental health professionals are working together proactively to ensure the transparency of the information and enhance the general public mental health resilience.

#### Information dissemination

To reduce panic and unwarranted health anxiety among the general public triggered by fake news disseminated through social media, China’s central health authority holds daily press conferences to provide people and mass media with accurate and reliable data on the number of infected people, the number of deaths and updated scientific knowledge of preventing COVID-19. Mental health education is also incorporated in the daily updates of COVID-19, so as to increase public awareness of mental health issues associated with the pandemic. Besides, the general public can also get access to real-time, accurate and accessible information about the epidemics through multiple official websites or internet and mobile-device applications.

#### Public education

Online mental health education on preventing COVID-19 infection is available freely on multiple online platforms. More than 40 books, hundreds of popular science articles and numerous educational videos have been published which target population groups with different levels of mental health needs (Level 1- Level 4 population), and special populations (children, pregnant women, the elderly, patients with mental disorders and/or major physical diseases, etc.). Most of the publications aim at promoting mental health resilience during the epidemic [18]. For example, the handbooks on psycho-education about mental illnesses associated with COVID-19 released by China’s National Health Commission (NHC) have different sections addressing unique mental health needs for different population groups [19].

#### Assessment and intervention

To provide mental health assessments and psychological counselling to the general public, hospital official websites and multiple popular applications offer online self-assessment tools for current mental well-being. Online consultation support is offered freely if respondents of these assessment tools score above the cut-off points. In addition, more than 300 mental health hotlines have been in operation since the outbreak to provide counselling services for the general public [20].

### Challenges and opportunities

In contrast to other natural disasters (disasters such as earthquake, fire, explosion, etc.), psychological intervention is difficult to be carried out in a standard face-to-face manner during the COVID-19 outbreak, especially in the most severely affected place — Wuhan. As telecommunication, telepsychology and telepsychiatry have widely expanded and replaced the traditional face-to-face consultation in the communities, the effectiveness of the current mental health support warrants further review and reflection.

#### Accessibility

The accessibility of mental health services is limited for the general public due to the relative shortage of mental health professionals in China. According to nationwide surveys during the COVID-19 epidemic, stress (35%), anxiety (34%) and depression (30%) were prevalent among the general population [9, 21]. However, there are only 2.19 registered psychiatrists and 5.51 psychiatric nurses among every 100,000 population in China [22, 23]. There is also a shortage of counselling psychologists (estimating 30,000 to 40,000 in China) [24], with a lack of an official system of accreditation, registration and licensing [25, 26]. In addition, the capacity of social workers is also limited in China with a total number of around one million, and few were qualified for delivering mental health services [27]. Compared with the population in need, mental health professionals are definitely in severe shortage. Apart from the shortage of mental health services providers, physical accessibility is also a major challenge. As most of the mental health professionals could not arrive in the “disaster site” at an early stage of the outbreak, residents who experienced difficulties in seeking medical care for themselves or their family members, and those who underwent grief and bereavement were not able to get adequate psychosocial support in time due to strict isolation. In addition, some special groups, such as the elderly or children, have limited access to smartphones or the internet [28]. It is difficult for them to benefit from online psychological counseling. Effort was made to enhance mental health competencies among primary care doctors and workers in community centers [11]. However, access to psychosocial support remains quite limited due to an absence of a clear preparedness plan for emergency social services, a lack of an overall coordination between different social agencies and strict isolation measures in many cities [29].

#### Acceptability

Notwithstanding the high prevalence of mental health needs, we also notice that few people actively seek mental health care. For example, based on interviews with telepsychology units based in hospitals and universities, few people called for mental health services in the past couple of months [30, 31]. Some people might take the initiative to ask for help online, but online counseling may not be effective for all those with needs and may occasionally bring about secondary trauma due to inability of “being there” and “knowing something” [32]. Technical and logistic problems encountered by mental health providers during online counseling have been provided in previous reports [20]. Poor mental health literacy and stigma associated with mental illnesses might contribute to the relatively low utilization of mental health services [23, 33].

## Mental health support in the designated hospitals

### Overview of the strategies

As for in-reach mental health support for in-patients, on February 21th, 2020, the Joint Prevention and Control Mechanism of the State Council issued an emergency response plan for the deployment of a national psychological rescue team to Wuhan [34]. This was a step-up effort to meet the ever-increasing mental health needs of those infected and hospitalized. The national psychological rescue team recruited over 400 mental health professionals from eight other provinces and was further divided into local rescue teams, with each team of 5–10 mental health professionals providing care to one medical unit in Wuhan (As of February 2020, there were 53 designated hospitals for patients with moderate and severe symptoms and 16 sheltered hospitals for patients with mild symptoms in Wuhan). With each hospital now having its own designated local psychological rescue team, the mental health needs of hospital workers and in-patients can be assessed and addressed in a more assertive way than before.

#### Training and mental health services for healthcare workers

To address the mental health needs of Level 1 population (infected patients and their healthcare workers), healthcare workers preparing for frontline clinical work are provided with brief mental health training to equip them with the basic knowledge and skills in identifying and referring in-patients in need of further mental health care. The training also provided some self-help techniques to enhance their mental health resilience in face of psychological stress associated with care of patients with severe infections. Besides, mental health professionals were working in full capacity to provide support for frontline hospital workers. Notifications were delivered regularly to inform them of the available mental health care, including group relaxation practice, online mindfulness training sessions, one-on-one counseling, etc.

#### Mental health services for patients

Newly admitted patients are provided with booklets educating them about common mental health issues associated with COVID-19 and ways of coping with these mental health needs. In addition, various kinds of therapeutic group activities have been integrated into daily ward routines with an aim to alleviate loneliness, boredom and frustration brought about by the infection as well as the prolonged quarantine periods. Due to strict isolation measures in these isolation wards, mental health professionals provide psychological counseling for infected patients mainly through online and telephone means. For patients with serious mental health risks like suicidal attempts or major behavioural disturbances, face-to-face interviews are then conducted by a rescue team member with appropriate personal protective equipment.

### Challenges and opportunities

While the above work does help to minimise the adverse psychological effects on some patients and healthcare workers, there is still much room for improvement. Since February 24th, 2020, the authors have stationed in Wuchang Fangcang hospital and Tongji Hospital in Wuhan City. As one of the local psychological rescue teams based in designated hospitals in Wuhan, we were responsible for mental health education and training, psychological intervention and psychiatric consultation in the wards. Based on our work experience, the accessibility and acceptability of in-reach mental health care are even more complicated in designated hospitals than that of public mental health care for the general and other at-risk groups.

#### Accessibility

During our work, we found that patients who had experienced trauma before hospitalization (such as those who were unable to get medical treatment on time, having a sense of suffocation and near-death experience related to their unattended COVID-19 infections, or witnessing the death of loved one) were those who experienced severe psychological stress even after the recovery of COVID-19 infection. However, they rarely expressed their psychological pain in emotional terms but rather complained about having residual physical symptoms related to the COVID-19 infection. In addition, most healthcare workers seldom focused on patients’ psychological stress even though patients’ negative emotions might have been expressed in the form of irritability or anxiety. Most frontline healthcare workers lacked experience in taking care of people with mental health needs and therefore might not be aware of or feel not well-equipped to assess these needs. The most common management being offered for such distress was by prescribing sedatives such as benzodiazepines, as we found that nearly 10% of patients in our medical infection ward units were prescribed with a benzodiazepine. It was our observation that many of these patients were traumatized but were not provided with evidence-based psychological interventions.

#### Acceptability

Although the National Health Commission attached great importance to mental health support, the local psychological intervention team was not very “welcomed” in hospitals on their arrival [35]. Patients in isolation wards of the designated hospitals were willing to accept psychological assessments and further interventions as needed. However, many patients in the shelter hospitals were not willing to receive mental health assessments or evidence-based mental health interventions as indicated. They were more engaged in generic group activities such as square dance, Tai Chi or other relaxation training activities. Chinese traditional psychotherapy was also applied to some patients. Many hospital staff were also reserved about seeking mental health care themselves even when they experienced significant mental health problems. It was only after they have witnessed significant improvement in mental health among their patients that they became more willing to participate in therapeutic group activities and other kinds of mental health interventions. According to our documentation, among the 2400 healthcare workers in Tongji Hospital, 7% of them attended regular evening online training which consisted of mindfulness breathing, muscle relaxation training, and self-healing hypnosis; 13% attended in-hospital group relaxation practice; 10% reported insomnia and received group counseling and/or pharmacotherapy; only 0.5% sought one-on-one psychological counseling.

## Reflections on China’s mental health support in response to COVID-19

How to resolve the dilemma between the high prevalence of mental health needs and the low acceptability of mental health care? In order to overcome these challenges, the authors propose the following reflections and strategies so as to raise mental health awareness of the whole society, to improve the structure and mechanism of mental health responses towards public emergencies and disasters, and to maximise the effectiveness of current mental health support work.

### The “universality” of mental health support during public health emergencies

Since public health emergencies like SARS and COVID-19 epidemic affect every citizen, the “universality” of mental health support should involve every sector of society. From the beginning of the outbreak, mental health professionals in China have actively taken up the role of mental health education, training and intervention, as well as the management of mental disorders related to COVID-19. This has contributed to preventing and minimizing adverse psychological consequences during the epidemic. However, such work is still regarded as activities within the premise of mental health care. The concepts of ‘no health without mental health’ and ‘parity of physical and mental health care’ are far from being well embraced in China. Therefore, in order to improve accessibility, acceptability, and effectiveness in addressing people’s mental health needs, it is important to include mental health support as an integral part in every aspect of infection outbreak prevention and control work, from political decision-making bodies to mass media, from patients to the general public, from public health promotion to all branches of medicine, and from a nation to the whole world.

Firstly, fighting with the “infodemic” requires the joint effort of the whole society [36, 37]. Inconsistent or ambiguous information, and over-exposure to adverse news can trigger widespread panic and anxiety in the public as well as undermine the confidence of front-line workers [38–41]. As such, the government and social media should work together to ensure that information released to the public is appropriate, scientific, authoritative and practical. Besides, the authorities and health professionals should clarify misconceptions and dispel myths in time, and recommend appropriate coping strategies in face of the infodemics. The legislative bodies should impose penalties on those who spread rumours that significantly cause significant disruptions in society [42]. The public should be educated to seek reliable information from authoritative channels and to remain calm and clear-minded about the massive influx of conflicting information. This will hopefully reduce the sense of confusion and loss, as well as adverse psychological stress caused by the ‘infodemic’ [36, 37, 43].

Secondly, mental health support should be integrated into the daily life of the general public. As the online mental health services have spatial limitations and may not provide sufficient support, psychosocial support (i.e., community resources and primary care settings) should be well-coordinated and actively provide services in the communities. The residents can strengthen social support in their communities by helping and encouraging each other during the epidemic. Tailored practical tips to help specific populations to build up mental health resilience should be shared online and disseminated as widely as possible. TV and radio programs could broadcast mental health related tips and skills at a fixed time every day, such as breathing and muscle relaxation exercises, body scan and mindfulness exercises. Only by integrating the knowledge and skills of mental health care into everyone’s daily life can mental health resilience be built up to alleviate the stress caused by the epidemic and prevent subsequent mental health problems.

Thirdly, mental health support should be integrated as part of all different medical specialties. Studies have shown that good clinical care, such as empathic listening to patients’ concerns, clear explanation of treatment plans and provision of emotional support, not only reduces the occurrence of adverse psychological consequences in patients’ and carers’ [44–46], but also prevents clinicians from burnout [47]. Therefore, mental health curriculum such as enhancing empathy and communication skills should be structurally embedded in health professionals’ training programs. Besides, mental health professionals should be included as members of multidisciplinary teams caring for different physical health needs, so that the mental health needs of both patients and care staff can be addressed in time. In addition, through collegial collaboration, mental health care could be embedded in the standard care packages of all healthcare workers, so as to enhance the acceptability and accessibility of mental health intervention. For example, if a patient receives psychological support from his or her long-term and trusted healthcare providers, the outcome may be far better than that provided by an unfamiliar psychotherapist. Only by integrating mental health care into daily medical care can the accessibility and acceptability of mental health support for patients and healthcare workers be continually improved.

Fourthly, a joint effort to establish international collaboration is needed to address the mental health challenges caused by global public health emergencies such as the COVID-19 pandemic. It includes sharing one’s own healthcare experiences among different nations and institutions, providing updates to enrich the psychological crisis intervention guidelines prepared by the World Health Organization, providing mental health training and education specific for COVID-19 by member countries with relatively more clinical expertise to those members deprived of mental health capacities through international professional associations like World Psychiatric Association, and conducting large-scale international epidemiological surveys.

### The “timeliness” of mental health support for public health emergencies

The timeliness of mental health support for public health emergencies needs appropriate strategies during prevention and preparedness, response, and recovery (PPRR). After the unexpected SARS outbreak in 2003–2004, the Chinese Central Government formulated and eventually published the national preparedness plan for public health emergencies in 2006 [48]. However, the preparedness plan did not include specific details about what and how mental health and psychosocial services should be organised and delivered. In 2013, the China’s National Mental Health Law proposed that the emergency response plans formulated by the national or provincial government should include mental health support [49]. The mental health law puts forward the mechanism of mental health support in response to public emergencies (i.e., central organization by the government and coordination between multiple departments). It also emphasizes that mental health education for the public should permeate schools, families, workplaces and the community. In August 2019, a few months just before the outbreak, a special team under the China’s Association for Disaster and Emergency Rescue Medicine (CADERM) was established — the mental health rescue team [50]. The rescue team recruited mental health experts experienced in psychological intervention for public emergencies all over the country and aimed to develop comprehensive preparedness plans for different kinds of disasters. Unfortunately, the preparedness plan of mental health in response to a large-scale epidemic has not been fully established by the time of the infectious outbreak [51]. Besides, there is still a severe shortage of mental health professionals to provide support for public emergency events in China [29]. With the lessons learned from the COVID-19 epidemic, a more comprehensive mental health support system in response to different emergency events needs to be established in China. Relevant national guidelines and specific mental health training programs for coping with natural and man-made disasters should be provided for health professionals, policymakers, social workers, community volunteers and mass media. As each region might have different levels of resources and resilience for emergency events, every provincial-level and town-level region in China should develop a practical preparedness plan for emergencies under the national guideline. Psychological intervention teams for emergency events should be established according to the mental health needs of regions. With different stakeholders being equipped with the knowledge and skills about mental health emergencies, different departments can then work together as a team to provide comprehensive mental health support in response to emergencies. At the same time, general public education on enhancing mental health literacy and mental health resilience should be carried out routinely. With a more specific preparedness plan established before a disaster, a more comprehensive, well-coordinated and timely response can then be promulgated at an early stage of an emergency. All these efforts will improve accessibility, acceptability and effectiveness of mental health support at the time of any outbreak.

During the response period, the mental health support system should be established timely based on the preparedness plan, with each stakeholder having a detailed work plan and a clear reporting and governance structure. In particular, for mental health professionals, each local psychological crisis intervention centre should come up with an emergency plan for the deployment of different psychological crisis intervention teams, so that resource-constrained areas can still have access to mental health services. In addition, structured supervision and evaluation system should be established in place to ensure the quality of mental health services. Regular clinical experience sharing and exchanges between different intervention teams are needed for enriching and enhancing clinical knowledge and skills accumulated from clinical work in different cities, as well as increasing sense of cohesion among the teams. Given the geographical, ethnic and religious diversities in China, mental health professionals working in different provinces should have been trained with relevant cultural mental health competencies. With culturally relevant mental health interventions being available in some cities, the acceptability of mental health care will then be greatly enhanced.

In the aftermath of an epidemic, most mental health support teams will evacuate from the front-line clinical teams. Yet it is expected that mental health problems may become more prominent after the end of the epidemic, especially in worst-affected areas (i.e., Wuhan and Hubei Province in this COVID-19 epidemic). Grief and bereavement, post-traumatic stress disorders, anxiety disorders, and depression are common mental health problems associated with disasters. From the perspective of psychiatry, more attention and effort are needed in support of mental health after the epidemic than during the epidemic. Coping with mental health need challenges after the disaster should never be underestimated.

### The “scientific rigour” of mental health support during public health emergencies

Mental health support includes acute psychological stress relief work, psychological crisis intervention, and the acute and long-term management of mental disorders. Specifically, mental health resilience is essential for preventing and recovery from mental health problems. It is important for the public to learn self-help techniques for reducing mental distress during the pandemic. The general public should be entitled to receiving authoritative and scientific health information for protecting self and others, maintaining a healthy lifestyle and learning ways of positive thinking to tide over challenging times. If these self-help strategies cannot alleviate psychological distress, people should consider seeking further evidence-based psychological interventions.

The goal of crisis intervention is to minimise negative emotions and restore daily functioning. For mild psychological distress, psychological intervention serves to alleviate excessive negative emotions. For more severe mental distress, psychological crisis intervention aims at preventing further deterioration and facilitates further referral to professional psychiatric services. In order to provide evidence-based and effective crisis intervention during such mental health emergencies, the authors have the following recommendations. First, timely and accurate mental health assessment should be provided. Psychological stress needs to be proactively assessed in the most affected populations, as well as other at-risk groups as appropriate. Second, alleviation of distressing negative emotions should take precedence over challenging negative cognitions, as crisis intervention is time-limited and should focus on prompt relief of the most distressing aspects of presenting mental health problems. As such, evidence-based and easily learned emotion management techniques like muscle relaxation, breathing techniques, creating personal safe spaces and self-compassion, and emotional first-aid kits should be taught. These are short-term strategies which, if clinically indicated, should be followed by additional evidence-based cognitive-behavioural strategies like exposure therapy or imagery rescripting for post-traumatic stress disorders delivered by the next tier of expert mental health professionals. Third, at a later stage of the epidemic, the focus of mental health support should be stress-related disorders brought about by the epidemic. For instance, sense of loss of good health and loved ones, sense of alienation and marginalisation by others are two frequent difficulties faced by patients recovering from the COVID-19. Fourth, therapeutic benefits of online mental health services during public health emergencies need to be evaluated. Although some studies suggest no significant differences in clinical outcomes between smartphone-based intervention groups and face-to-face professional clinical intervention groups [52], these studies were not delivered during public health emergencies and hence might not be generalisable to such situations. As such, the effectiveness and side-effects of online psychological interventions during public health emergencies is a top priority research question. More evidence also needs to be accrued to understand how these interventions, if confirmed to be safe and efficacious, can be disseminated widely to the public and be accepted as culturally relevant and appropriate treatment approaches.

## Conclusion

COVID-19 pandemic has created an immense adverse impact on the physical and mental health of the population in China. Its aftermath will leave a strong scar on the mental health of people who have lost their good physical health, their loved ones and their faith in personal and world safety. However, the outbreak also provides us with an opportunity to reflect on how we should enhance our public mental health emergency responses in preparation for another large-scale natural or man-made disaster in the coming future.

## Data Availability

There are no data in this work.
